# Intrathecal Use of Isobaric Levobupivacaine 0.5% Versus Isobaric Ropivacaine 0.75% for Lower Abdominal and Lower Limb Surgeries

**DOI:** 10.7759/cureus.8373

**Published:** 2020-05-31

**Authors:** Priyank Samar, Sarla Pandya, Tanvi A Dhawale

**Affiliations:** 1 Anesthesiology, K.J. Somaiya Medical College and Hospital, Mumbai, IND

**Keywords:** sensory block, motor block, onset of motor block, onset of sensory block, spinal anesthesia

## Abstract

Background

This study was undertaken to compare and evaluate the efficacy of 3-ml 0.5% isobaric levobupivacaine versus 3-ml 0.75% isobaric ropivacaine in patients undergoing elective lower abdominal and lower limb surgeries.

Methods

We allocated 60 patients into two groups (n=30 each) to receive either a spinal block of 3-ml 0.5% isobaric levobupivacaine (group L) or 3-ml 0.75% isobaric ropivacaine (group R). Haemodynamic parameters were measured intraoperatively till the end of surgery and postoperatively for two hours. The onset and duration of sensory block and motor block were recorded. Adverse events were also recorded. The student’s unpaired t-test was used for comparing the continuous variables.

Results

The mean age in group L was 37.83 ±16.51 years and the mean age in group R was 38.50 ±12.97 years. The mean onset of sensory block in group L (6.97 ±1.82 mins) was significantly faster than in group R (8.47 ±2.55 mins), p<0.05. Similarly, so was the mean onset of motor block in group L (10.27 ±1.92 mins) versus group R (12.93 ±2.55 mins), p<0.05. The mean duration of sensory block in group L (147.63 ±27.53 mins) was significantly longer than in group R (97.40 ±12.38 mins), p<0.05, as was the mean duration of motor block in group L (207.33 ±22.27 mins) versus group R (146.60 ±21.22 mins), p<0.05. In group L, 13.3% of patients had complications, with hypotension being the most common (6.7%); in group R, 40% had complications, of which bradycardia was the most common (13.3%).

Conclusion

There was an earlier onset of sensory and motor block and prolonged duration of sensory and motor block with intrathecal administration of 3-ml 0.5% isobaric levobupivacaine as compared to 3-ml 0.75% isobaric ropivacaine. Haemodynamic parameters were more stable with levobupivacaine than ropivacaine. Adverse effects were more common with ropivacaine.

## Introduction

Lower abdominal and lower limb surgeries may be performed under regional (spinal, epidural, or both) or general anaesthesia. Bupivacaine 0.5% heavy was the only drug used for spinal anaesthesia after the discontinuation of intrathecal use of lidocaine. However, its cardiotoxic and central nervous system side effects have led to the development of its pure S (-) enantiomers: ropivacaine and levobupivacaine [1-3]. Ropivacaine is a long-acting amide local anaesthetic agent that is less lipophilic than bupivacaine and less likely to penetrate large myelinated motor fibers, resulting in a relatively reduced motor blockade [1,2]. Levobupivacaine is an S (-) enantiomer of the long-acting local anaesthetic bupivacaine, having less cardiotoxic and central nervous system effects in comparison with bupivacaine [3]. Clinically, levobupivacaine is well tolerated in a variety of regional anaesthesia techniques both after bolus administration and continuous postoperative infusion. Reports of toxicity with levobupivacaine are scarce and occasional toxic symptoms are usually reversible; yet, levobupivacaine has not entirely replaced bupivacaine in clinical practice [4]. Clinical studies show no significant differences in onset, duration and sensory block, but complete regression of sensory block takes longer [5-7]. The regression of motor block occurs earlier with levobupivacaine and ropivacaine as compared to bupivacaine [8]. Although levobupivacaine and ropivacaine were introduced a few years ago, to our knowledge, there are very few studies on the use of isobaric levobupivacaine 0.5% and isobaric ropivacaine 0.75% for spinal anaesthesia for obstetric, abdominal and orthopaedic surgeries, and levobupivacaine has been found to be more potent* *[6-14]. Also, there have been very few studies among the Asian population [13,15]. Current literature on the use of these drugs focuses mostly on epidural and labour analgesia and peripheral nerve blocks [15-18].

This study was undertaken to compare and evaluate the efficacy of 3-ml 0.5% isobaric levobupivacaine versus 3-ml 0.75% isobaric ropivacaine for level, onset, duration of sensory and motor blockade of spinal anaesthesia, haemodynamic changes and safety in American Society of Anesthesiologists (ASA) class 1 and II adult patients undergoing elective lower abdominal and lower limb surgeries among an Asian population.

## Materials and methods

After gaining Institutional Review Board and Ethics Committee approval (Thesis/142373/2014/11445) and obtaining written informed consent from participants, we conducted a review of prospectively collected data related to 60 adults who underwent lower abdominal and lower limb surgical procedures under spinal anaesthesia at a single centre. Inclusion criteria were as follows: subjects between the age of 18-70 years, ASA status I and II, weight range of 40-90 kg, and posted for lower abdominal and lower limb surgeries. Patients who were ASA status III and IV, pregnant and lactating patients, those with a history of bleeding disorders, those who were allergic to local anaesthetics, patients on anticoagulants, those suffering from infection at the site of spinal needle insertion, those having spinal abnormalities like spina bifida, meningocele or those who refused to give consent were excluded.

In total, 60 adult patients scheduled to undergo elective surgery and satisfying all the inclusion criteria were enrolled in the study, after receiving written informed consent. They were randomly allocated into two groups (n=30 each) according to computer-generated random numbers using the sealed envelope technique, to receive either a spinal block of 3-ml 0.5% isobaric levobupivacaine (group L) or 3-ml 0.75% isobaric ropivacaine (group R). The anaesthesia attending/resident observer was not blinded to the drug administered.

After confirming adequate fasting status, and premedication with injection glycopyrrolate 0.2 mg (0.004 mg/kg) intramuscular (i.m), baseline parameters of heart rate, systolic blood pressure (SBP), diastolic blood pressure (DBP) and mean blood pressure (MBP) were noted. At our institution, we follow the protocol of administering injection glycopyrrolate prior to regional anaesthesia to prevent vasovagal event and prefer it over injection atropine as it causes relatively less tachycardia. Intravenous access was secured with 18 G cannula in non-dominant hand and injection ondansetron 4 mg was given intravenously and Ringer's lactate was started at 2 ml/kg/hr of fasting as a preloading solution before the intrathecal block. Intravenous fluids were given as per kg body weight and operative loss. Spinal anaesthesia was given under aseptic precautions - group L: 3 ml of 0.5% levobupivacaine (isobaric); group R: 3 ml of 0.75% ropivacaine (isobaric).

The following parameters were studied - haemodynamic parameters: pulse rate, SBP and DBP and arterial oxygen saturation (SpO2) were measured at baseline and intraoperatively at 0, 2, 5, 10, 15, 20, 25, 30 mins and thereafter every 15 mins till the end of surgery and postoperatively for two hours at 0, 30, 60, 90 and 120 mins; the onset of sensory block: the time interval between intrathecal administration of the drug and maximal spread of sensory block; the onset of motor block: the time interval between intrathecal administration of the drug and achievement of Bromage score 3; duration of sensory block: the time elapsed between injection of the drug and 2-dermatome regression of anaesthesia from the maximum sensory level; duration of motor block: the time elapsed between injection of the drug to point in which Bromage score is back to 2.

Adverse events were recorded. Hypotension, 20% fall below baseline SBP, was treated with injection ephedrine hydrochloride 6-mg intravenous bolus. Bradycardia, heart rate below 50, was treated with a titrated dose of atropine 0.1-0.6-mg intravenously. Hypoxia, Spo2 <95%, was treated with supplemental oxygen via face mask. Nausea, vomiting, dry mouth, dizziness, headache, respiratory depression and shivering were recorded. Injection ondansetron 0.04 mg/kg was given intravenously for nausea. Injection tramadol 50 mg along with ondansetron 0.04 mg/kg was given intravenously for shivering.

Statistical analysis

Apriori analysis was performed by keeping the confidence limits at 95% and the power of study at 80% to detect a minimum of 10% difference in the degree of sensory/motor blockade between the two groups; the minimum sample size required was 25 in each group. Thirty patients were included in each group for better validation. Descriptive analysis of numerical data (mean ±SD) and categorical data (frequency and percentage) was performed. Statistical tests like student’s unpaired t-test were used for continuous variables as per normality distribution of data using SPSS Statistics software v.19 (IBM, Armonk, NY), and a p-value of <0.05 was considered statistically significant.

## Results

The mean age in group L was 37.83 ±16.51 years and that in group R was 38.50 ±12.97 years. The mean weight of patients in group L was 66.80 ±9.21 kg and that in group R was 61.27 ±11.59 kg. Demographic data in both groups were comparable.

Mean baseline pulse rate in group L was 83.1 ±10.16 beats per minute (bpm) and that in group R was 87.17 ±11.88 bpm, and it decreased at two mins after intrathecal injection to 71.6 ±9.37 (group L) and 72.4 ±11.14 bpm (group R), but the difference between two groups was not statistically significant (p>0.05). The mean pulse rate intraoperatively at each time interval in the two groups is shown in Table 1. The difference was not significant (p>0.05). Both the fall and the subsequent rise in mean pulse rate in group L was more gradual as compared to the steep fall and rise in group R, although not statistically significant (p>0.05). Mean pulse rate postoperatively fluctuated within a narrow range and the difference was not significant (p>0.05) except at 120 mins when the mean pulse rate was higher in group R (85.07 ±10.05 bpm) than group L (80.07 ±5.28 bpm), which was statistically significant (p<0.05; Table 2).

**Table 1:** Comparison of mean pulse rate at various postoperative periods between study groups *Statistically significant SD: standard deviation

Time (mins)	Group L, bpm (mean ±SD)	Group R, bpm (mean ±SD)	P-value
0	82.00 ±3.96	82.97 ±6.46	>0.05
30	82.53 ±3.40	84.80 ±6.44	>0.05
60	81.67 ±4.23	84.97 ±8.63	>0.05
90	80.73 ±5.02	83.13 ±16.58	>0.05
120	80.07 ±5.28	85.07 ±10.05	<0.05*

**Table 2:** Comparison of mean intraoperative pulse rate between groups *P<0.05 statistically significant SD: standard deviation

Time (mins)	Group L, bpm (mean ±SD)	Group R, bpm (mean ±SD)	P-value*
Baseline	83.10 ±10.16	87.17 ±11.88	>0.05
0	81.33 ±8.88	86.10 ±12.05	>0.05
2	71.60 ±9.37	72.40 ±11.39	>0.05
5	75.07 ±5.55	71.67 ±10.91	>0.05
10	75.33 ±5.26	74.40 ±7.24	>0.05
15	76.50 ±5.68	76.57 ±7.10	>0.05
20	77.60 ±6.73	77.60 ±6.75	>0.05
25	78.87 ±6.28	78.27 ±6.82	>0.05
30	78.53 ±4.66	78.00 ±7.48	>0.05
45	80.36 ±6.30	78.60 ±7.93	>0.05
60	79.88 ±5.37	79.07 ±7.91	>0.05
75	79.61 ±2.89	81.05 ±6.15	>0.05
90	82.89 ±5.32	81.35 ±7.40	>0.05
105	82.29 ±4.63	80.64 ±5.83	>0.05
120	83.64 ±4.92	81.11 ±5.30	>0.05
135	83.17 ±5.00	81.20 ±5.40	>0.05
150	81.50 ±2.84	83.20 ±5.40	>0.05
165	81.50 ±2.82	90.00 ±5.40	>0.05

The baseline SBP in group L was 122.93 ±10.04 mmHg and that in group R was 125.30 ±13.39 mmHg. Intraoperative SBP demonstrated a greater fall from the baseline in group R (100.03 ±14.07 mmHg) compared to group L (108.73 ±9.38 mmHg) at two mins, and this was also seen at subsequent time intervals (Table 3). The difference in mean intraoperative SBP between two groups intraoperatively at 2, 5, 10, 15, 20, 25, 30, 45, 60, 75 and 90 mins was statistically significant (p<0.05) with a lower mean SBP recorded in group R. The difference in mean intraoperative DBP between two groups at each interval was not significant (p>0.05) (Table 3). There was no significant difference in the SBP and DBP in the two-hour postoperative period, except for mean post-operative DBP at 60 and 120 mins (Table 4). There were no significant differences in the MBP. At all intervals intraoperatively and postoperatively, Sp02 saturation was found to be >95% in both the groups.

**Table 3:** Comparison of Intraoperative blood pressure between groups *Statistically significant SBP: systolic blood pressure; DBP: diastolic blood pressure; MBP: mean blood pressure; SD: standard deviation

Time (mins)	Group L SBP, mmHg (mean ±SD)	Group R SBP, mmHg (mean ±SD)	P-value	Group L DBP, mmHg (mean ±SD)	Group R DBP, mmHg (mean ±SD)	P-value	Group L MBP, mmHg (mean)	Group R MBP, mmHg (mean)
Baseline	122.93 ±10.04	125.30 ±13.39	>0.05	80.93 ±4.57	83.23 ±8.34	>0.05	94	97
0	122.07 ±8.85	121.10 ±12.38	>0.05	80.13 ±4.54	81.37 ±7.88	>0.05	94	95
2	108.73 ±9.38	100.03 ±14.07	<0.05*	67.67 ±2.04	68.20 ±7.72	>0.05	81	79
5	109.27 ±7.12	102.70 ±11.01	<0.05*	70.73 ±3.34	71.87 ±7.00	>0.05	84	82
10	110.40 ±6.72	106.03 ±9.70	<0.05*	72.77 ±4.66	73.60 ±6.99	>0.05	85	84
15	112.40 ±8.98	106.87 ±9.00	<0.05*	73.33 ±4.34	74.20 ±5.75	>0.05	86	85
20	114.07 ±7.88	107.40 ±8.99	<0.05*	75.27 ±5.15	73.20 ±13.89	>0.05	88	85
25	116.87 ±9.58	107.83 ±7.69	<0.05*	76.20 ±4.55	75.73 ±5.93	>0.05	90	86
30	116.73 ±8.98	110.80 ±8.33	<0.05*	77.00 ±4.48	76.20 ±6.15	>0.05	90	88
45	116.71 ±6.64	110.60 ±9.50	<0.05*	77.29 ±5.14	77.83 ±5.75	>0.05	90	89
60	119.08 ±6.90	115.50 ±8.61	<0.05*	79.00 ±5.62	78.43 ±5.89	>0.05	92	91
75	123.22 ±7.39	118.48 ±8.50	<0.05*	80.89 ±2.58	80.14 ±5.81	>0.05	95	93
90	123.89 ±6.30	119.16 ±8.20	<0.05*	80.11 ±2.22	80.50 ±5.61	>0.05	95	93
105	123.43 ±6.48	120.00 ±6.38	>0.05	80.29 ±2.33	80.36 ±6.78	>0.05	95	93
120	123.86 ±3.63	120.45 ±7.63	>0.05	79.00 ±2.80	79.91 ±6.45	>0.05	94	93
135	127.33 ±2.14	121.20 ±7.56	>0.05	78.83 ±1.99	83.20 ±7.95	>0.05	95	96
150	125.92 ±4.90	122.50 ±9.57	>0.05	80.33 ±7.77	80.50 ±7.72	>0.05	96	94
165	120.00 ±4.00	136.00 ±7.42	>0.05	74.00 ±5.65	90.00 ±7.65	>0.05	89	105

**Table 4:** Comparison of postoperative blood pressure between groups *Statistically significant SBP: systolic blood pressure; DBP: diastolic blood pressure: MBP: mean blood pressure; SD: standard deviation

Time (mins)	Group L SBP, mmHg (mean ±SD)	Group R SBP, mmHg (mean ±SD)	P-value	Group L DBP, mmHg (mean ±SD)	Group R DBP, mmHg (mean ±SD)	P-value	Group L MBP, mmHg (mean)	Group R MBP. mmHg (mean)
0	127.40 ±4.64	124.67 ±8.26	>0.05	81.40 ±4.10	81.77 ±3.82	>0.05	97	96
30	127.87 ±5.11	125.93 ±6.11	>0.05	81.73 ±5.37	82.27 ±4.89	>0.05	97	97
60	126.67 ±5.18	126.00 ±5.11	>0.05	78.93 ±4.54	82.87 ±4.83	<0.05*	95	97
90	126.83 ±4.95	125.57 ±4.85	>0.05	80.07 ±5.13	82.37 ±4.15	>0.05	96	97
120	126.47 ±4.05	126.10 ±4.67	>0.05	80.33 ±4.39	83.00 ±4.62	<0.05*	96	97

The mean onset of sensory block in group L (6.97 ±1.82 mins) was significantly faster than in group R (8.47 ±2.55 mins), p<0.05, as was the mean onset of motor block in group L (10.27 ±1.92 mins) versus group R (12.93 ±2.55 mins), p<0.05 (Table 5). The mean duration of sensory block in group L (147.63 ±27.53 mins) was significantly longer than in group R (97.40 ±12.38 mins), p<0.05, as was the mean duration of motor block in group L (207.33 ±22.27 mins) versus group R (146.60 ±21.22 mins), p<0.05 (Table 5).

The complications encountered in both groups are shown in Table 6. Of the 30 patients in group L, 13.3% had complications, with hypotension being the most common (6.7%) followed by nausea and shivering. Out of the 30 patients in group R, 40% had complications, of which bradycardia was the most common (13.3%) followed by hypotension, nausea and shivering. Both bradycardia and hypotension were found in 3.3%. None of the patients in group L had bradycardia.

**Table 5:** Mean onset and duration of motor and sensory block in both groups *Statistically significant SD: standard deviation

Onset	Group L, time in mins, (mean ±SD)	Group R, time in mins, (mean ±SD)	Significance (unpaired t-test)
Mean onset of sensory block	6.97 ±1.82	8.47 ±2.55	<0.05*
Mean onset of motor block	10.27 ±1.92	12.93 ±2.55	<0.05*
Mean duration of sensory block	147.63 ±27.53	97.4O ±12.38	<0.05*
Mean duration of motor block	207.33 ±22.27	146.60 ±21.22	<0.05*

**Table 6:** Complications in both groups

Complication	Group L		Group R	
	n	%	n	%
Bradycardia	-	-	4	13.3
Hypotension	2	6.7	3	10.0
Hypotension and bradycardia	-	-	1	3.3
Nausea	1	3.3	2	6.7
Shivering	1	3.3	2	6.7
Total complications	4	13.3	12	40

## Discussion

The reduced lipophilicity of ropivacaine is associated with decreased potential for central nervous system toxicity and cardiotoxicity when compared to bupivacaine; the lower lipid solubility of ropivacaine would mean that it is likely to produce a greater block of sensory and motor function than bupivacaine [3].

Onset and duration of sensory block

We observed that the mean onset of the sensory block with levobupivacaine (6.97 ±1.82 mins) was significantly faster than with ropivacaine (8.47 ±2.55 mins), p<0.05, and the mean duration of the sensory block with levobupivacaine (147.63 ±27.53 mins) was significantly longer than with ropivacaine (97.40 ±12.38 mins), p<0.05. Mantouvalou et al. reported that the time to achieve maximum surgical analgesia with 3 ml of 0.5% isobaric levobupivacaine was 11 ±6 mins [9]. Fattorini et al. reported that the time to achieve maximum surgical analgesia with 3 ml of 0.5% isobaric levobupivacaine was 12 ±6 mins [10]. Wahedi et al. found that the maximum onset of analgesia in 3 cc of 0.75% isobaric ropivacaine was 13 mins [12]. D’Souza et al. reported that the onset of the sensory block with 3 ml of 0.5% levobupivacaine was 5.50 ±4.25 mins, and it was 5.25 ±4.00 mins with 3 ml of 0.75% ropivacaine [13]. Mantouvalou et al. reported that the 2-segment regression time of sensory blockade with 3 ml of 0.5% levobupivacaine was 65 ±11 mins [9]. Wahedi et al. reported that time taken for sensory block regression from maximum T8 to T10 with 3 ml of 0.75% glucose-free spinal ropivacaine was 50 mins [9].

Onset and duration of motor block

In our study, the mean onset of motor block was quicker with levobupivacaine (10.27 ±1.92 mins) versus ropivacaine (12.93 ±2.55 mins), p<0.05, and the mean duration of motor block was longer with levobupivacaine (207.33 ±22.27 mins) as compared to ropivacaine (146.60 ±21.22 mins), p<0.05. D’Souza et al. found that the median onset of Bromage 3 with 0.5% levobupivacaine (isobaric) was five mins, and it was 18 mins with 0.75% ropivacaine (isobaric), which was statistically significant [13]. Mantouvalou et al. and Fattorini et al. found that the onset of Bromage 3 with 0.5% isobaric levobupivacaine was 11 ±7 mins and 11 ±6 mins respectively [9,10]. Wahedi et al. reported that the time taken to achieve Bromage score 3 with 0.75% glucose-free spinal ropivacaine was 15 mins [12]. D’Souza et al. found that the median duration of Bromage 3 motor block with 0.5% levobupivacaine (isobaric) was 240 mins, and it was 195 mins with 0.75% ropivacaine (isobaric) [13]. Fattorini et al. found that regression of motor block from Bromage 3 to 2 in the 0.5% levobupivacaine (isobaric) group was 256 ±6 mins [13]. Mantouvalou et al. found that regression of motor block from Bromage 3 to 2 with 0.5% levobupivacaine (isobaric) was 79 ±19 mins [9]. Wahedi et al. found that the duration of the motor block in the 0.5% levobupivacaine (isobaric) group was 260 mins [12].

Haemodynamic parameters

Both the fall and the subsequent rise in mean pulse rate intraoperatively with levobupivacaine was more gradual as compared to the fall and rise with ropivacaine; however, it was not statistically significant. The steeper rise in pulse rate with ropivacaine at two hours suggested early wearing off of the subarachnoid block. SBP demonstrated a greater fall from the baseline intraoperatively in group R compared to group L until 90 mins. The SBP in both groups reached the lowest value at approximately the same time around two mins. However, steeper rise in mean SBP in group R at 165 mins suggested early wearing off of subarachnoid block with ropivacaine. Results suggest that the haemodynamic effects of levobupivacaine are relatively more stable than the more labile effects of ropivacaine.

Complications

Among the 60 patients studied, no complication was found in 44 patients (73.3%). Hypotension was documented in five patients (8.3%) followed by bradycardia in four patients (6.7%), nausea in three patients (5%) and shivering in three patients (5%). Complications were lower in patients who received levobupivacaine (13.3%) as compared to those who received ropivacaine (40%). Bradycardia was found only in group R (4%) while one patient (3.3%) in group R had both bradycardia and hypotension. Coppejans and Vercauteren compared the effects of spinal levobupivacaine with bupivacaine for Caesarean section and found a lower incidence of hypotension with the S-enantiomer levobupivacaine [14]. Nausea and shivering were observed more commonly in patients who received ropivacaine (6.7%) compared to those who received levobupivacaine. Mantantouvalou et al. found a 10% incidence of nausea in patients who received 3 ml of 0.5% levobupivacaine [9].

More recent literature discusses the use of ropivacaine and levobupivacaine for epidural and obstetric analgesia and local peripheral nerve blocks [15-18]. Kumar et al. found that the onset and duration of epidural analgesia were shorter with ropivacaine than levobupivacaine [15]. Li et al. found that levobupivacaine is more potent than ropivacaine when used for peripheral nerve blocks [17].

Limitations

The main limitation of this study was the heterogeneity of surgical procedures. The observer was not blinded to the drug administered. Our study involved validating previously described findings in an Asian population. A larger prospective double-blinded study with a single surgical procedure is recommended to evaluate this further.

## Conclusions

In this study, we compared and evaluated the efficacy of 3-ml 0.5% isobaric levobupivacaine versus 3-ml 0.75% isobaric ropivacaine in patients undergoing elective lower abdominal and lower limb surgeries. We can conclude that there is an earlier onset of sensory and motor block and prolonged duration of sensory and motor block with intrathecal administration of 3-ml 0.5% isobaric levobupivacaine. Haemodynamic parameters are more stable with levobupivacaine. Adverse effects are more common with ropivacaine.
